# The ITA.LI.CA Staging System: A Novel Staging System for Hepatocellular Carcinoma

**DOI:** 10.1371/journal.pmed.1002005

**Published:** 2016-04-26

**Authors:** Neehar D. Parikh, Amit G. Singal

**Affiliations:** 1 Department of Internal Medicine, University of Michigan, Ann Arbor, Michigan, United States of America; 2 Department of Internal Medicine, UT Southwestern Medical Center, Dallas, Texas, United States of America; 3 Harold C. Simmons Cancer Center, UT Southwestern Medical Center, Dallas, Texas, United States of America

## Abstract

In this Perspective, Neehar Parikh and Amit Singal discuss the advantages of the ITA.LI.CA staging system for prognoses of liver cancer developed by Alessandro Vitale and colleagues.

Hepatocellular carcinoma (HCC) is an increasingly common and highly morbid malignancy. Appropriate tumor staging is crucial to inform prognosis and guide treatment decisions in clinical practice; however, staging for HCC can be complex because of many patient-level factors that can impact prognosis and treatment eligibility. HCC is a unique malignancy in that it typically occurs in the setting of underlying organ dysfunction (i.e., cirrhosis), so most staging systems take both tumor burden and the degree of underlying liver dysfunction into account for prognostication. The most commonly used staging systems for HCC include the Barcelona Clinic Liver Cancer (BCLC) system [1], the Hong Kong Liver Cancer (HKLC) system [2], the Cancer of the Liver Italian Program (CLIP) system [3], the Japan Integrated Staging (JIS) system [4], and the Model to Estimate Survival in Ambulatory HCC patients (MESIAH) (Table 1) [5]. However, there has been a lack of consensus regarding the optimal staging system, and none is universally accepted. Most existing systems were derived from single-center data, lack prospective or external validation, and lack granularity in intermediate- and advanced-stage patients. In this issue of PLOS Medicine, Alessandro Vitale and colleagues detail the derivation and validation of a novel staging system for HCC, the ITA.LI.CA system [6]. The ITA.LI.CA system attempts to address many deficiencies of prior staging systems and demonstrates better discriminant ability for predicting survival than existing HCC staging systems in both internal and external validation cohorts.

**Table 1:** Existing staging systems for hepatocellular carcinoma.

Staging System	Components	Derivation Cohort
ITA.LI.CA [6]	Tumor burden	Multicenter Italian cohort
	Child-Pugh score	
	Functional status	
	AFP	
Barcelona Clinic Liver Cancer [1]	Tumor burden	Single center European cohort
	Child-Pugh score	
	Functional status	
Hong Kong Liver Cancer [2]	Tumor burden	Single center Chinese cohort
	Child-Pugh score	
	Functional status	
Cancer of the Liver Italian Program [3]	Tumor morphology	Multicenter Italian cohort
	AFP	
	Presence of portal vein thrombus	
Japan Integrated System [4]	TNM Staging	Multicenter Japanese cohort
	Child-Pugh score	
Model to Estimate Survival in Ambulatory HCC Patients [5]	Age	Single center US cohort
	MELD score	
	Serum albumin	
	Largest tumor diameter	
	Number of tumors	
	Presence of tumor vascular invasion	
	Extrahepatic metastases	
	AFP	

Abbreviations: AFP, alpha fetoprotein; MELD, Model For End-Stage Liver Disease; TNM, TNM Classification of Malignant Tumours

The ITA.LI.CA system was derived from a prospective multicenter database of over 5,000 HCC patients from Italy. The majority of patients in the cohort had hepatitis C infection, nearly all (97%) had good performance status, and three-fourths had well-compensated cirrhosis. External validation was performed using data from a Taiwanese cohort of over 2,600 patients, with the primary etiology of liver disease for patients in this cohort being chronic hepatitis B infection. Using a priori variable selection based on prior staging systems and a literature review, the authors derived a model that uses a prognostic score based upon tumor burden (categories of 0, A, B1–3, and C), functional status, Child-Pugh score, and alpha fetoprotein (AFP) concentration (≤1,000 or >1,000 ng/ml). The model had better discriminant ability than any of the existing staging systems in the training, internal validation, and external validation cohorts (c-statistic values being 0.72, 0.71, and 0.78, respectively).

The BCLC staging system is currently the most widely accepted staging system and has been endorsed by the American Association for the Study of Liver Disease (AASLD) and European Association for the Study of the Liver (EASL) [7,8]. Though some aspects of the ITA.LI.CA system are rooted in the BCLC, it is distinct in several important ways: first, in subclassifying BCLC stage B patients into B1, B2, and B3 categories based on degree of intrahepatic tumor burden; second, in differentiating patients with intrahepatic and extrahepatic metastases; and finally, by incorporating the serum biomarker AFP. In the BCLC system, all patients with liver-isolated disease, without metastases or vascular invasion, are grouped together as BCLC stage B [1]. However, differential survival and locoregional treatment allocation for BCLC stage B patients has been demonstrated in several studies [9,10]. For example, distinguishing whether BCLC stage B patients are within (B2) or beyond (B3) Milan criteria is important when considering liver transplantation. Similarly, recent data suggest prognosis in patients with extrahepatic metastases is worse than those with intrahepatic metastases, so the differentiation in the ITA.LI.CA system, essentially subclassifying the BCLC stage C patients, adds further granularity to estimating prognosis [11]. Finally, AFP is not part of the BCLC staging system but can serve as a surrogate for occult vascular invasion, distant metastases, or aggressive tumor biology. Patients with an AFP > 500 ng/ml have a higher risk of recurrence post-transplant as well as a lower likelihood of response to locoregional therapy [12]. These three important distinctions as compared to the BCLC system likely explain, in part, the higher prognostic accuracy of the ITA.LI.CA staging system in derivation and validation cohorts.

Although the model demonstrated good prognostic discrimination among study patients, it should be noted that most patients in both cohorts had good performance status, compensated cirrhosis, and early or intermediate stage tumors. It is unclear if the ITA.LI.CA staging system would perform as well in cohorts with high rates of hepatic decompensation, poor performance status, and/or advanced tumor stage—subgroups that currently account for the majority of HCC patients in several countries, including the United States. Further, very few patients in this study—less than 2% in the derivation cohort and none in the external validation cohort—underwent liver transplantation, a curative therapy for both the tumor and underlying cirrhosis that plays a crucial role in the management of HCC patients.

Potential future steps in further refinement and validation of the ITA.LI.CA staging system include prospectively assigning treatment allocation recommendations to patients in different stages and validation in more contemporary cohorts, in which transplantation or systemic therapies are utilized to a greater extent.

## Conclusions

The authors of the ITA.LI.CA staging system have introduced a novel staging system for HCC, building on existing staging systems. This system helps in better differentiation of intermediate and advanced stage patients, and the prognostic model contains several important factors that are clinically relevant in the care of patients with HCC. This system is an important iteration in the evolution of staging for HCC, and, while it enters a crowded field, the ITA.LI.CA staging system is a worthy entrant.

## Abbreviations

AASLD: American Association for the Study of Liver Disease
AFP: alpha fetoprotein
BCLC: Barcelona Clinic Liver Cancer
CLIP: Cancer of the Liver Italian Program
EASL: European Association for the Study of the Liver
HCC: hepatocellular carcinoma
HKLC: Hong Kong Liver Cancer
JIS: Japan Integrated Staging
MESIAH: Model to Estimate Survival in Ambulatory HCC patients

## References

[1] Llovet JM, Bru C, Bruix J. Prognosis of hepatocellular carcinoma: the BCLC staging classification. Semin Liver Dis. 1999;19(3):329–38. Epub 1999/10/13. 10.1055/s-2007-1007122 . 10518312

[2] Yau T, Tang VY, Yao TJ, Fan ST, Lo CM, Poon RT. Development of Hong Kong Liver Cancer staging system with treatment stratification for patients with hepatocellular carcinoma. Gastroenterology. 2014;146(7):1691–700.e3. 10.1053/j.gastro.2014.02.032 . 24583061

[3] A new prognostic system for hepatocellular carcinoma: a retrospective study of 435 patients: the Cancer of the Liver Italian Program (CLIP) investigators. Hepatology. 1998;28(3):751–5. 10.1002/hep.510280322 . 9731568

[4] Kudo M, Chung H, Osaki Y. Prognostic staging system for hepatocellular carcinoma (CLIP score): its value and limitations, and a proposal for a new staging system, the Japan Integrated Staging Score (JIS score). J Gastroenterol. 2003;38(3):207–15. 10.1007/s005350300038 . 12673442

[5] Yang JD, Kim WR, Park KW, Chaiteerakij R, Kim B, Sanderson SO, et al Model to estimate survival in ambulatory patients with hepatocellular carcinoma. Hepatology. 2012;56(2):614–21. 10.1002/hep.25680 22370914 PMC3564594

[6] Farinati F, Vitale A, Spolverato G, Pawlik TM, Huo T-l, Lee Y-H, et al Development and validation of a new prognostic system for patients with hepatocellular carcinoma. PLoS Med. 2016;13(4): e1002006. 27116206 10.1371/journal.pmed.1002006 PMC4846017

[7] European Association For The Study Of The L, European Organisation For R, Treatment Of C. EASL-EORTC clinical practice guidelines: management of hepatocellular carcinoma. J Hepatol. 2012;56(4):908–43. 10.1016/j.jhep.2011.12.001 . 22424438

[8] Bruix J, Sherman M, American Association for the Study of Liver D. Management of hepatocellular carcinoma: an update. Hepatology. 2011;53(3):1020–2. 10.1002/hep.24199 21374666 PMC3084991

[9] Bolondi L, Burroughs A, Dufour JF, Galle PR, Mazzaferro V, Piscaglia F, et al Heterogeneity of patients with intermediate (BCLC B) Hepatocellular Carcinoma: proposal for a subclassification to facilitate treatment decisions. Semin Liver Dis. 2012;32(4):348–59. 10.1055/s-0032-1329906 . 23397536

[10] Arizumi T, Ueshima K, Iwanishi M, Minami T, Chishina H, Kono M, et al Validation of a Modified Substaging System (Kinki Criteria) for Patients with Intermediate-Stage Hepatocellular Carcinoma. Oncology. 2015;89 Suppl 2:47–52. 10.1159/000440631 . 26584036

[11] Yoo JJ, Lee JH, Lee SH, Lee M, Lee DH, Cho Y, et al Comparison of the effects of transarterial chemoembolization for advanced hepatocellular carcinoma between patients with and without extrahepatic metastases. PLoS ONE. 2014;9(11):e113926 10.1371/journal.pone.0113926 25427152 PMC4245068

[12] Mehta N, Sarkar M, Dodge JL, Fidelman N, Roberts JP, Yao FY. Intention to treat outcome of T1 hepatocellular carcinoma with the "wait and not ablate" approach until meeting T2 criteria for liver transplant listing. Liver Transpl. 2016;22(2):178–87. 10.1002/lt.24360 . 26479422 PMC4803445

